# The World of Oral Cancer and Its Risk Factors Viewed from the Aspect of MicroRNA Expression Patterns

**DOI:** 10.3390/genes13040594

**Published:** 2022-03-26

**Authors:** Ovidiu Aghiorghiesei, Oana Zanoaga, Andreea Nutu, Cornelia Braicu, Radu Septimiu Campian, Ondine Lucaciu, Ioana Berindan Neagoe

**Affiliations:** 1 Department of Oral Health, “Iuliu Hatieganu” University of Medicine and Pharmacy, 15 Victor Babeș Street, 400012 Cluj-Napoca, Romania; ovidiu.agh@gmail.com (O.A.); ondineluc@yahoo.com (O.L.); 2 Research Center for Functional Genomics, Biomedicine and Translational Medicine, “Iuliu Hațieganu” University of Medicine and Pharmacy, 23 Marinescu Street, 400337 Cluj-Napoca, Romania; nutu.andreea@umfcluj.ro (A.N.); braicucornelia@yahoo.com (C.B.); ioana.neagoe@umfcluj.ro (I.B.N.); 3 Department of Oral Rehabilitation, “Iuliu Haţieganu” University of Medicine and Pharmacy, 15 Victor Babeș Street, 400012 Cluj-Napoca, Romania; rscampian@email.com

**Keywords:** oral cancer, miRNAs, risk factors, epigenetics

## Abstract

Oral cancer is one of the leading causes of death worldwide, with a reported 5-year survival rate of around 50% after treatment. Epigenetic modifications are considered to have a key role in oral carcinogenesis due to histone modifications, aberrant DNA methylation, and altered expression of miRNAs. MicroRNAs (miRNAs) are small non-coding RNAs that have a key role in cancer development by regulating signaling pathways involved in carcinogenesis. MiRNA deregulation identified in oral cancer has led to the idea of using them as potential biomarkers for early diagnosis, prognosis, and the development of novel therapeutic strategies. In recent years, a key role has been observed for risk factors in preventing and treating this malignancy. The purpose of this review is to summarize the recent knowledge about the altered mechanisms of oral cancer due to risk factors and the role of miRNAs in these mechanisms.

## 1. Introduction

**Figure 1:**
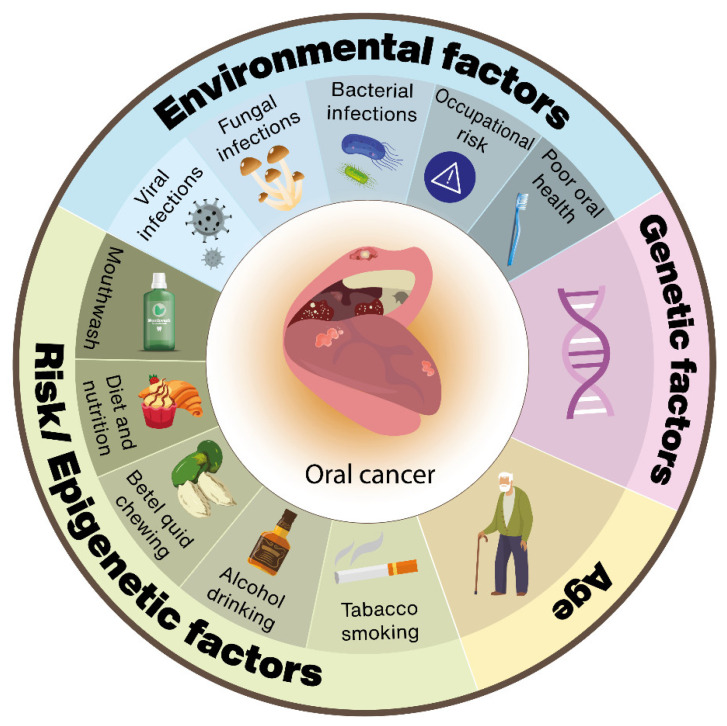
The link between the main factors related to oral carcinogenesis, including environmental, risk/epigenetic factors, genetic background, and age.

Oral cancer is a type of cancer in which tumors develop in the oral cavity, lip, tongue, gingiva, or oropharynx [1,2]. According to the World Health Organization, oral cancer is among the most prevalent cancers worldwide, representing the 16th most common malignancy and the 15th leading cause of death worldwide with an incidence rate higher in men than women; the risk of developing this disease is higher after the age of 45.3 [3,4]. The five-year survival rate can also be correlated with tumor advancement: about 80% for patients with stage I and 20% for patients with stages III or IV [5,6,7]. The different habits of the population, education, and access to medical services have a powerful influence on oral cancer incidence [8,9]. Oral squamous cell carcinoma (OSCC) is the most common type of oral cancer (approximate 90%); the remaining 10% of oral malignancies involve rare histologic subtypes (minor salivary gland carcinomas, lymphomas, and melanoma) [10,11,12,13]. Various risk factors have been notably associated with oral cancer [9,14,15,16,17], with the majority of oral cancers being related to the use of cigarettes, alcohol, and betel quids [11] (Figure 1).

Worldwide, tobacco or alcohol was associated with 72% (95% CI 61% to 79%) of HNC cases, of which 35% were attributed to tobacco and alcohol combined [18,19,20,21]. Worldwide, in 2018, 38,000 of 900,000 cases were attributable to the human papillomavirus (HPV) [22,23]. The most widely used predictors of survival for oral cancer are tumor stage at the time of presentation, as well as HPV genotypes that are present in the tumor, in conjunction with the tumor site and treatment procedure [24].

Growing evidence supports the role of epigenetic regulation in the process of oral carcinogenesis being considered an early event [25,26]. Epigenetic regulation is evaluated as an early event in the process of oral cancer development, epigenetic modifications including hypermethylation in the promoter region of genes, post-translational histone modifications, and post-transcriptional regulation by microRNAs [27]. In addition, early molecular alterations along with dysregulation of coding and non-coding genes have an important role in modulating the response to the therapy [28].

## 2. Major Risk Factors for Oral Cancer

### 2.1. Epigenetic Alteration Associated with Risk Factors

Epigenetics refers to modifications in DNA expression that do not involve alterations in the DNA sequence. The mechanisms responsible for these changes are DNA methylation, histone modification, and post-transcriptional gene downregulation by microRNAs (miRNA), which are able to induce overexpression of oncogenes but also the silencing of tumor suppressor genes [29,30]. The epigenetic mechanism of DNA methylation can lead to gene silencing in neoplasms; the process of DNA methylation consists of a covalent insertion of a methyl group into the 5 carbon (C5) position of cytosine to generate 5-methylcytosine (5-mC) in cytosine/guanine dinucleotide islands (CpGIs) [31]. CpGIs are often discovered in the promoter of some genes, including tumor suppressor genes and proto-oncogenes [31]. Epigenetic alterations are related to chromosomal instability and modifications in transcriptional control, affecting the gene expression differences that are found in human cancers [31]. Recent evidence has shown that promoter hypermethylation, especially in CpG islands, can enhance the downregulation of tumor suppressor genes, while the downstream signaling pathways are dysregulated [32].

In the DNA methylation event, a series of enzymes named DNA methyltransferases (DNMTs) with a role in the covalent addition of a methyl group to a CpG dinucleotide were found [33,34]. DNA methylation was observed in 28–58% of precancerous oral tissues, while in oral cancer a progressive increase in oral cancers has been found [35,36]. The use of DNA methylation should be mentioned as a highly predictive blood biomarker to detect heavy alcohol drinking [37]. Moreover, the hypermethylation of p16 is an early event found in patients with long-term tobacco use and premalignant lesions [35]. DNA methylation was demonstrated to be present in adjacent normal tissues as an early event and/or process of field cancerization occurring in the oral cavity [38]. An imbalance in tumor suppressor gene methylation status is related to multiple risk factors or environmental agents [39].

#### 2.1.1. Tobacco Consumption

Worldwide, tobacco is responsible for nearly 6.4 million deaths and hundreds of billions of dollars of economic damage each year; in 2012, the total economic cost of smoking was estimated to be PPP $1852 billion (US$1436 billion), representing 1.8% of the world’s annual gross domestic product (GDP) [40]. According to specialists’ estimates, by 2030, tobacco consumption will lead to the death of more than 8 million people each year, with most cases estimated to occur in developing countries with a lower income, low levels of education, and prevention programs [41]. The concentration and potency of carcinogens from tobacco depend on the type of tobacco product and its method of consumption [40]. Thus, more than 60 known carcinogens in smoking tobacco and about 16 in unburned tobacco have been identified [42]. A stronger association of smoking with larynx and pharynx cancer has been observed due to a higher exposure of the larynx and pharynx to smoke than the oral cavity [43,44]. Cigar smoking and smokeless tobacco use were associated with an increased risk for premalignant and malignant lesions of the oral cavity, as well as with periodontal diseases, tooth loss, and dental implant failures [45,46]. In recent years, smokeless tobacco has become more and more popular in developing countries. Smokeless tobacco consumption consists of direct contact with mucous membranes commonly in nasal snuff or placed in the oral vestibule, thus leading to a significant increase in the risk for squamous cell carcinoma [47]. Chewing tobacco is popular in Western Europe and North America, with the main types being plug, loose-leaf, and twist [48]. Additionally, moist snuff (ground tobacco) has become common in North America and Scandinavia, and the habit of oral snuff was associated with the so-called ‘snuff dipper’s cancer’ that is classically described as verrucous carcinoma [49]. The principle of duration, more important than frequency, has been demonstrated for smoking. Thus, smoking fewer cigarettes per day over a longer period proves to be more harmful to oral cancer risk than smoking more cigarettes per day over a shorter period [50]. The nicotine levels for heat-not-burn cigarettes are considered to be lower, and as a result, the health risks are unclear [51]. At the same time, carcinogens have been found in the heat-not-burn (HNB) tobacco aerosol, with the levels being lower than the smoke from ordinary cigarettes, but no current data are available regarding the risk of HNB cigarettes for the development of oral cancer [52]. Additionally, in vitro studies identified a lower effect of aerosol from HNB tobacco products compared to cigarette smoke [53,54,55]. Regarding snus use, an association with the presence of non-neoplastic oral mucosal lesions has been reported. Once the user has been stopped, the lesions are rapidly healed, and the health risks associated with snus are considerably lower than those associated with cigarette smoking [56].

The interaction between smoking and carcinogen metabolizing gene polymorphisms by modulating promoter methylation of tumor suppressor genes has been revealed [57]. Oxidative stress and persistent reactive species in tissues produce the occurrence of cell cycle-regulated mutations, disruption of the immune system [58], and abnormal expression of different epigenetic genes, such as p53, P13K, DAPK, GLUT-1, MGMT, and p16, in oral epithelium [41]. Several frequently mutated genes, including TP53, CASP8, and CDKN2A have been identified in OSCC tumors from subjects with different tobacco consumption habits. Furthermore, TP53 and HRAS revealed mutually exclusive mutation patterns [59].

Dibenzo [def,p] chrysene (DBP) has been found in tobacco and can be a potential biomarker for early detection of OSCC, with an important role in oral carcinogenesis due to the epigenetic alteration that occurred due to tobacco use [60,61]. In patients with an early history of smoking, a significant correlation was observed between promoter methylation of CDKN2a and tobacco carcinogens, smoking exposure influencing the promoter methylation in a gene-specific manner in HNSCC. Thus, the link between promoter methylation and an increase in tobacco carcinogen exposure may be a clue for the presence of cells resistant to gene alteration that is induced via mutations [61]. Chronic smoking and drinking can induce aberrant methylation in the P15 gene, an effector of transforming growth factor (TGF-β)-induced cell cycle arrest. The downregulation of P15 is essential in the oral carcinogenesis process and was also methylated in 64% of OSCC heavy smokers [62].

Tobacco and its metabolites can change the methylation profiles in oral cancer by altering the expression of DNA methyltransferase expression [63]. In oral cancers, the methylation profile of genes evidences a differential methylation pattern, involving global hypomethylation in the genome’s repeat sequences and hypermethylation of specific genes [64]. DNA methylation was also observed in the normal-appearing surgical margins of OSCC patients in which the incidence of local recurrences was highlighted. DAPK promoter hypermethylation was revealed in surgical margins to be linked to a decrease in overall survival [65]. An inverse significant association was demonstrated between tobacco consumption and DNA methylation, while the smoking effect is amplified, the overall methylation index is reduced, and cigarette smoke causes hypomethylation [39].

#### 2.1.2. Betel Quid/Nut Chewing

In many parts of South Asia, the high preponderance of oral cancer has been linked to the habit of betel quid chewing, one of the dominant etiological factors for the development of this malignancy and an important menace to public health [66,67]. Multiple factors were linked to malignant transformation in the oral mucosa of betel quid chewers; the involvement of each component in betel quid was individually, synergistically, and coordinately found in the carcinogenesis process [67]. Thus, the carcinogens from alcohol and tobacco can act synergistically in this process besides betel quid; an increase in the duration for the habit of betel quid chewing can generate chronic inflammation of the oral cavity with numerous ulcerations and microbiome dysbiosis [67]. Furthermore, these multiple factors intertwine and can cause malignant transformation in the oral mucosa of betel quid chewers [67]. Approximately 10% of the world’s population chews betel nuts regularly, being the fourth most commonly used psychoactive substance in the world [68] [69]. Recent evidence showed the association of oral cancer with betel quid-areca nut habit, but also with oral premalignant lesions, such as oral submucous fibrosis and leukoplakia, both with the capacity to develop malignant transformation [70]. The RARB gene was hypermethylated in the OSCC tongue tissues from a mouse model cotreated with arecoline and 4-nitroquinoline-1-oxide (4-NQO). Moreover, in the human oral cancer cells, gene reexpression was identified by 5′-aza-2′-deoxycytidine (5-aza-dC) at 2 μM. Thus, de novo DNA methyltransferases proved to be associated with the gene epigenetic alternations of OSCC [71]. Areca nut components play key roles in the pathogenesis of betel quid-induced oral cancer via induction of ROS, IL-1α, EGF/EGFR, JAK, and COX signaling pathways. In addition, these components cause aberration in cell cycle- and differentiation-related proteins of oral keratinocytes [72].

#### 2.1.3. Alcohol Consumption

Alcohol consumption turned out to be the leading risk factor for disease burden worldwide, responsible for nearly 10% of global deaths among populations aged 15–49 years [73]. Nowadays, the role of alcohol in the development of oral cancer has been proven due to its potential to cause malignancies of the oral cavity, esophagus, larynx, pharynx, and liver [49]. The risk of oral cancer is considerably increased by the association of alcohol consumption with tobacco [49]. Alcohol has a major role in the DNA methylation process and histone modifications that increase carcinogenesis due to the two major components—ethanol and its metabolite, acetaldehyde. DNA methylation can also be affected by alterations in folate metabolism and transmethylation reactions [74].

Alcohol can be directly related to 4.2% of cancer deaths [75] and 26.4% of all lip and oral cavity cancers worldwide [76], with the main metabolites of ethanol being considered a class 1 carcinogen [77]. These metabolites are directly involved in the carcinogenesis process by the occurrence of the disturbance of DNA synthesis and repair, the development of DNA adducts, and DNA hypomethylation that leads to the alteration of oncogene expression [78]. Furthermore, alcohol metabolites have an indirect solvent role, causing more mucosa permeability to other carcinogens, such as those from tobacco [79]. The effect of drinking alcohol was associated with a higher risk for oropharyngeal and hypopharyngeal cancer than for laryngeal cancer among those that have never smoked [80].

In a study conducted by Lubin, using the INHANCE dataset, it was found that higher alcohol drinks/day over a shorter period can be more harmful than fewer alcohol drinks/day over a longer period [50]. Studies regarding the interaction between genetic polymorphisms in genes that code for alcohol metabolizing enzymes and alcohol consumption have revealed that the prevalence of these genetic polymorphisms varies by ethnicity [81,82]. Smoking cigarettes has been linked to a two-fold increased risk of oral cancer among those that have never consumed alcohol, with the risk being observed to increase with prevalence, persistence, and pack-years of cigarette smoking [83]. The combined effects of tobacco and alcohol use lead to a higher risk for the development of oral cancer compared to multiple individual effects [18,84]. It is well known that ethanol from wine is oxidized to form acetaldehyde in the oral cavity [85]. Acetaldehyde is a metabolite with genotoxic properties that leads to the overexpression of oncogenes but can also cause the silencing of tumor suppressor genes [86]. Moreover, ethanol and acetaldehyde cause alteration of methyl transfer during carcinogenesis, generating DNA hypomethylation with alteration in the expression of oncogenes and tumor-suppressor genes and stimulating tumor cell dissemination [85]. In oral keratinocytes exposed to alcohol and acetaldehyde, the dysregulation of lncPSD4-1 and in-NETO1-1, two key long non-coding RNAs strongly linked to the development of head and neck squamous cell carcinoma, has been observed [87]. Oral cancer cell proliferation was also increased by the expression of miR-30a and miR-934, which promote the induction of anti-apoptotic gene Bcl-2 [88].

#### 2.1.4. Diet and Nutrition

An important role in the development of oral cancer has been assigned to dietary and nutritional habits, increased consumption of citrus fruits and raw vegetables (especially yellow, green, and cruciferous vegetables), which are involved in the decrease of oral cancer risk [89,90,91,92] due to their regulation of the activity and the expression of transcription factors, growth factors, mediators of the inflammatory process, and intermediates of the cell cycle [93]. The International Head and Neck Cancer Epidemiology (INHANCE) Consortium, according to the results from 22 case-control studies, reported an odds ratio (OR) of 0.52 for high fruit consumption and 0.66 for high vegetable consumption [91]. The risk for oral cancer can increase with the consumption of red meat more than once a week compared to white meat (chicken, fish) [89]. Low consumption of fruit and vegetables or high consumption of meat along with increased exposure to tobacco and alcohol has been associated with a 10- to 20-fold increase in risk for development of oral cavity and pharyngeal cancer [90,94]. A case-control study from Italy reported a 50% decrease in oral carcinogenesis due to the dietary consumption of flavonoids [95]. Epigallocatechin-3-gallate (EGCG) inhibited cell proliferation and promoted apoptosis and autophagy in OSCC cells by inhibiting TP53, CASP8, and MYC [96]. Glucoraphanin and its bioactive metabolite sulforaphane promoted the detoxification of carcinogenic chemical agents found in tobacco smoke and NRF2-independent dephosphorylation/inactivation of pSTAT3, which is a crucial oncogenic factor in head and neck squamous cell carcinoma [97]. Consumption of minimally processed foods has been reported as a protective factor in the development of squamous cell carcinoma of the head and neck [98]. An in vivo mouse model demonstrated that a high-fat diet and male sex contribute to the pathology of 4-nitroquinoline-1-oxide-induced oral cancer [99]. An important dietary risk factor for the development of OSCC is the high intake of iron linked to the involvement of this element in major cellular processes, such as metabolism, cell growth, and proliferation, with the generation of nitrogen compounds and free radicals, which cause cell damage [100]. Obesity is also an important risk factor for OSCC; a high-fat diet (HFD) significantly accelerated oral carcinogenesis by recruitment and functional enhancement of myeloid-derived suppressor cells (MDSCs) [101].

#### 2.1.5. Mouthwash

A possible association between the use of mouthwash with alcohol and the risk of oral cancer is supported by a few epidemiological studies with contradictory results [102,103]. However, the development of oral cancer can be influenced by the use of mouthwash with alcohol simultaneously with other risk factors, such as tobacco or alcohol [104].

### 2.2. Environmental Factors

#### 2.2.1. Viral Infections

Persistent infections with human papillomavirus (HPV) lead to the development of cancer, with the majority of HPV-induced carcinomas being related to type 16 [105]. HPV infection is a major risk factor for HNSCC [106]. A worrying increase has been observed in the worldwide incidence of HPV-positive oropharyngeal cancer, especially among younger men in the United States and other Western countries [107,108]. Head and neck squamous cell carcinoma (HNSCC) and oral squamous cell carcinoma (OSCC) represent 3% and 2% of all malignant neoplasms in men and women, respectively [107,109]. Furthermore, HPV infection has been associated with other risk factors, such as open mouth (deep) kissing, number of sexual partners, number of oral sex partners, alcohol, and tobacco [110]. HPV infection has also been especially observed in wild-type TP53 tumors [59]. HPV-16 is the most common HPV type, responsible for ~90% of HPV-associated OSCCs, the other HPV types being HPV-18, HPV-33, and HPV-35 [111,112,113]. 84% and 61.5% of patients with OSCC were found to be positive for HPV and HPV-16 [114]. “High-risk” HPV (HR HPV), such as HPV-16 and 18, have been discovered in up to 99% of cervical carcinomas [115]. It has been reported that approximately 4% of patients with cervical HPV infection are co-infected with the same HPV types in the oral region [116].

Epstein-Barr virus was found to be associated with the development of nasopharyngeal carcinoma and oral squamous cell carcinoma [117,118], a meta-analysis of 53 studies reported a 2.5 increased risk for developing OSCC in patients with EBV infection [119]. Latent membrane protein-1 (LMP-1) is an important marker of most EBV-related malignancies, including for OSCC [120], LMP-1 activates multiple signaling pathways, such as NF-κB, JAK-STAT, JNK–p38, and PI3K–AKT [121].

Severe acute respiratory syndrome coronavirus 2 (SARS-CoV-2) infection is more threatening in cancer patients, mainly due to the aggressiveness of the tumor type but also due to the side effects of the cancer treatment [122,123]. In the case of this infection, the oral mucous membrane may be targeted by the virus due to the highly expressed angiotensin-converting enzyme 2 (ACE2), the main host cell receptor of the SARS-CoV-2 [124]. Aphthous-like and superficial necrosis are oral ulcerations that occur in patients diagnosed with COVID-19 [125]. However, additional studies are needed to investigate whether oral ulcerations are directly caused by SARS-CoV-2 infection or whether oral lesions are a coincidental event, as well as the progression of the lesions.

#### 2.2.2. Fungal Infections

*Candida albicans* is an opportunistic fungus that becomes pathogenic in immunocompromised individuals [126], is found to be an independent risk factor in the development of oral carcinoma [127]. Oral cancer occurrence was significantly associated with *Candida* oral colonization. Furthermore, the genotypic diversity of *C. albicans* strains is directly involved in oral carcinogenesis [127].

#### 2.2.3. Bacterial Infections

*Porphyromonas gingivalis* promoted the invasion and metastasis of highly invasive oral cavity cancers [128,129] by stimulation of matrix metalloproteinase and by apoptosis of activated T cells [128,130,131]. *Streptococcus anginosus* has also been associated with the carcinogenesis of oral cancer [132]. A positive association has been discovered between the capacity of oral *Candida* to metabolize alcohol to acetaldehyde and, thus, to promote oral cancer development [133].

#### 2.2.4. Occupational Risks

Socioeconomic status (SES), including educational level, income, and occupation, was linked to oral cancer incidence in many studies [10,134,135]. Commonly, the risk of the development of oral cancer is lower in persons with higher education and income levels. However, the risk for the development of oral cancer has been remarkably reduced by the progress made in the last period [136]. Multiple exposures to asbestos and polycyclic aromatic hydrocarbons have been strongly associated with the risk of oral and pharyngeal cancer [137]. One of the most common occupational and carcinogenic agents for oral cancer pathogenesis is wood dust [138]. Exposure to excessive solar radiation/ultraviolet (UV) light can cause lip cancers and may also produce actinic cheilitis, which may transform to OSCCs [49]. In medium-low-income countries, occupational exposures are less controlled and more frequent due to the lack of automatic monitoring equipment and self-protection in the work [139]. Access to dentists and oral cancer screening services was also associated with delays in establishing a diagnosis and poorer survival [140].

#### 2.2.5. Poor Oral Health

Poor oral hygiene is strongly associated with oral cancer, especially in association with other risk factors, such as tobacco and alcohol. A case-control study of Indian patients aged 18–45 years reported poor hygiene for 79% of the patients with oral cavity and oropharynx cancer [141]. The risk for the development of oral cancer is decreased by 26% by dental visits [142]. The Chinese population has observed a positive association between the number of missing teeth due to poor hygiene and head and neck cancer risk [143]. Detection of oral cancer cases at early stages can be accomplished by oral cavity screening, with the survival rates being improved if the disease is treated in early stages [144].

### 2.3. Genetic Factors

The role of Family History of Cancer (FHC) in oral cancer incidence led to contradictory opinions. Thus, some researchers believe that there is no evidence of a clear hereditary trait for oral cancers, except for Cowden syndrome (correlated with a few reported cases of head and neck cancer) and dyskeratosis congenital, a rare genetic disorder characterized by oral white lesions in young people, and with risk of transformation to cancer [145]. However, the familial risk for oral cancer has been assessed in several epidemiological studies suggesting a possible correlation between FHC and oral cancer [146,147,148,149]. The Utah Population Database resource, including about 1 million individuals, has been used for a unique comprehensive population-based study of familial cancer, with a standardized incidence ratio of oral cancer of 1.8 (95%) for patients with a first-degree relative with the same cancer type [149].

### 2.4. Age

Young age is known to be an independent factor for survival. Regarding oral malignancies, in young adults, oral cancer is a rare disease that is increasing in women [150]. In the United States, the incidence rate was found to increase in patients aged 55–64 years, with a median age of 63 years, and most of them being diagnosed at age 45 and above https://seer.cancer.gov/statfacts/html/oralcav.html (accessed on 19 December 2021). A population-based study in Taiwan reported that oral cancer patients with an age less than 45 years had a lower risk of mortality compared to middle age (45–65 years) or old age (>65 years) patients [3].

## 3. Oxidative Stress and Chronic Inflammation Associated with Risk Factors in Oral Cancer

Common risk factors, such as tobacco, alcohol, and betel nut/areca quid chewing, promote carcinogenesis via ROS-based mechanisms by the increase of oxidative DNA damage that is secondary to ROS generation [151]. The link between increased ROS levels and oral cancer development has been observed in tobacco chewers and smokers [41,152]. Tobacco smoke supports the production of ROS via the production of NO, superoxide, and hydrogen peroxide in different types of head and neck cancer [153]. Tobacco chewing and smoking can increase oxidative stress along with the increase of lipid peroxidation and oxidative DNA alteration, which can disturb antioxidant protection inducing the malignant process [154]. Chewing areca nuts is another risk factor for oral carcinogenesis by ROS generation; the increase in ROS levels leads to oxidative DNA damage [155]. Areca nut extract decreased cisplatin toxicity of OSCC cells by inducing autophagy through the AMPK/mTOR pathway and with remarkable increases in ROS levels [156]. The upregulation of MKP-1, increases in autophagy, and defense against apoptosis are also processes observed as pathogenesis-promoting mechanisms [157]. For betel quid, the major component is arecoline, which can promote the generation of reactive oxygen species (ROS) [68]. Patients with a history of areca quid chewing have been observed a ROS-induced overexpression of the Snail family of transcription factors, which can also be linked to metastasized lymph nodes [158,159]. In both normal and malignant cells, autophagy induction by the 30–100 kDa fraction of areca nut occurred through the production of reactive oxygen species [160]. The high levels of ROS, which leads to lipid peroxidation and binds to DNA to form mutagenic adducts, were correlated with chronic alcohol consumption and can generate single nucleotide polymorphisms in Cytochrome P450 2E1 (CYP2E1) CYP2E1 [161]. Studies regarding the effects of the EBNA1 protein of the Epstein–Barr virus on the redox pathway found that overexpression of EBNA1 is linked to the presence of increased levels of ROS and NADPH oxidases NOX1 and NOX2, which can generate ROS, being directly involved in the development of NPC [162]. Changes in cellular metabolism by high-risk HPV E2 proteins are important in carcinogenesis by inducing the Warburg effect, the localization of HPV-18 E2 at mitochondrial membranes promoting ROS release, and an increase in glycolysis [163]. Human papillomavirus type 16 E6 * is involved in the oral mutagenesis process by increasing oxidative stress due to a decrease in cellular antioxidant activity through the production of high levels of ROS and DNA damage [164]. HPV16 E6 and E7 proteins generated a chronic oxidative stress response via NOX2, promoting genomic instability and increased sensitivity to DNA damage in head and neck cancer cells [165]. The increased levels of ROS are generated by circCDR1as upregulation, which plays a major role in the activation of autophagy under a hypoxic microenvironment in OSCC, also supporting the role of ROS as a cellular autophagy regulator by suppression of mTOR pathway activation [166]. Furthermore, the inhibition of autophagy in combination with circCDR1as can be considered a potential therapeutic strategy for oral cancer [167].

Chronic inflammation induces epigenetic and transcriptomic modifications, and the chronic inflammation associated with reactive oxygen species represents an important source of DNA damage that is involved both in the development of oral carcinogenesis and in cancer treatment [41]. Tobacco and alcohol consumption, along with chronic inflammation, are important risk factors associated with dysregulations in the epigenetic pattern [168,169]. The chronic exposure of human mucosal epithelial cells to carcinogens from tobacco is related to the hypermethylation of different tumor suppressor genes [41,170]. Epigenetic alterations of key genes linked to the regulation of the DNA methylation feedback process were identified to maintain normal cell division in oral cell lines. In addition, a significant upregulation of CTLA4 was observed. In addition, the expression or promoter DNA methylation of CD28, a T cell activation promoter, did not suffer significant alterations [171].

## 4. The Functions of miRNAs Associated with Risk Factors in Oral Cancer

MicroRNA (miRNAs) are small non-coding RNA molecules with 18–25 nucleotides in length, playing important roles in biological processes, such as cell growth, proliferation, and apoptosis. The role in cancer progression is defined through post-transcriptional modification of gene expression and/or translational repression [172,173,174,175]. Furthermore, alteration of miRNA expression is crucial in cancer for clinically prognostic cancer, influencing the expression of many protein-coding genes [176,177,178]. A key role in the field of drug development is played by the detection of toxicity-related biomarkers and by the influence-related transcriptomics signals [179]. MiRNAs were highlighted in OSCC, making them potential candidates for screening and diagnosis, the association of these markers with OSCC being studied in primary tumors, but also in biopsies, serum, and saliva [180,181,182,183].

Studies regarding the discovery of new prognostic biomarkers for oral cancer to improve patient stratification accuracy revealed that increased miR-155 expression is a positive predictor of survival; this effect was strongly correlated with high CD8+ TIL numbers. Moreover, miR-185 was independently associated with decreased survival [184].

miRNAs are associated with multiple aspects of oral cancer, and epigenetic and alteration of the expression levels being emphasized [185]. miR-21 and miR-155 were the most studied miRNAs in OSCC; miR-155e-5p facilitated tumor progression and was found to be significantly upregulated in OSCC tissues and cell lines [186]. The overexpression of miR-21 in OSCC has been demonstrated in several studies [187,188,189]. MiR-770 was identified as an oncomiR, leading to a more prominent OSCC metastasis [190]. miR-1237 was found to be significantly overexpressed in OSCC tumor samples, especially in the early stages (stages I and II), and was associated with poor prognosis of OSCC patients [191]. miR-205 was found to be significantly downregulated in oral cancer, its overexpression reducing cell viability and inducing cell apoptosis by activation of caspase-3/caspase-7 [192].

**Figure 2:**
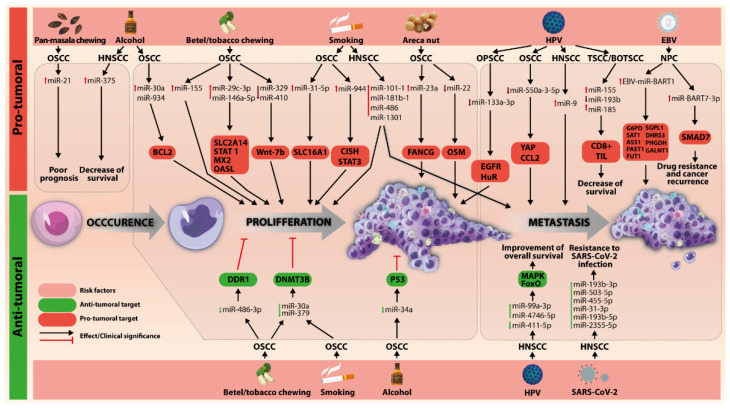
The connection between miRNAs and the main risk factors in oral cancer.

The miRNA altered pattern was linked to risk factors of oral carcinoma (Table 1, Figure 2).

In oral cancer, miRNA expression was found to be closely related to DNA methylation and hypermethylation, and CpG hypermethylation is associated with the downregulated expression of miR-203, miR-34b, miR-193a, and miR-137 in oral cancer cell lines [193]. Additionally, DNA hypermethylation is associated with a decrease in the expression of miR-218 and miR-585, and it is known that miR-218 can act as a suppressor of the mechanistic target of the rapamycin (mTOR)–Akt signaling pathway and independently of the phosphoinositide 3-kinase (PI3K)–Akt signaling pathway. Rictor is a possible target of miR-218 and was found to be upregulated in OSCC, which supports oral carcinogenesis by the epigenetic alteration of miR-218 and activation of the mTOR–Akt signaling pathway [194]. The proliferation and invasion of OSCC are promoted by the epigenetic alteration of miR-329 and miR-410, contributing to Wnt-7b overexpression and triggering the Wnt-β-catenin signaling pathway [195]. miR-200/miR-205 are miRNAs suppressed in disease with poor prognosis, being activated by epigenetic DNA hypermethylation in CD44 high OSCCs [196]. The presence of miR-31-mediated post-transcriptional regulation of SIRT3 in OSCC has been associated with OSCC progression, the oxidative stress in oral cancer being increased by SIRT3-miR-31 targeted to suppress mitochondrial activity. Additionally, the disturbance of the miR-31-SIRT3 cascade and the occurrence of metabolic aberrances are related to the development of OSCC [197]. The methylation in the p16 promoter was significantly higher in tobacco and maras powder users compared to those that have never used [35].

miR-218, miR-137e, miR-596, and miR-193a expression are significantly influenced by DNA methylation and loss of tumor suppressor activity and are downregulated in oral cancer [193,194,198].

### 4.1. miRNAs Altered by Epigenetic Risk Factors

The use of smokeless tobacco-like maras powder proved to increase the expression of miR-138 and miR-31 and decreased expression of miR-200b, miR-145, miR-375, miR-10b, miR-372, miR-92a, and miR-378a [218]. Tobacco chewing has been associated with an increase in miR-155 and a decrease in miR-542 expression, while tobacco smoking was linked to a decrease in miR-375, miR-23a, miR-203a, miR-23b, and miR-200b expression [199]. In pan-masala chewers, overexpression of miR-21 has been found; the increase in miR-23a and miR-155 expression was correlated with areca nut chewing, and alcohol consumption with overexpression of miR-375, miR-34a, and miR-183 [68,219]. miR-155 was found to have high levels in the tumor samples obtained from tobacco/betel quid chewers compared to those from individuals without this habit, with aberrant expression being due to exposure of oral mucosa to environmental factors, such as tobacco [187]. The overexpression of miR-23a was associated with the areca nut-chewing habit in oral cancer patients, and it is known that areca nut extract (ANE)-induced miR-23a was significantly associated with human malignancies [207].

Alcohol consumption in OSCC patients has been associated with high levels of miR-34a; downregulation of miR-34a and miR-143 may indirectly inhibit p53, revealing an indirect mode of p53 suppression in oral cancer [205]. Regarding miR-21 expression, an important relationship has been found between pan-masala chewers in OSCC patients, acting as a potential biomarker for early diagnosis, treatment, and prognosis [209]. An important upregulation of miR-31-5p and overexpression of miR-29c-3p and miR-146a-5p were observed in chewing tobacco-treated cells [199]. Recent evidence reported the dysregulation of miRNAs, such as miR-30a and miR-379, in oral cancer as a result of exposure to tobacco smoking and betel quid chewing; these miRNAs can regulate the retinoic acid pathway by targeting DNA methyltransferase B [200].

Chou et al. investigated the role of discoid domain receptor-1 (DDR1) tyrosine kinase and miR-486-3p as potential therapeutic targets of oral cancer as a result of exposure to betel nut alkaloids. Thus, a low level of miR486-3p is involved in OSCC tumorigenesis through DDR1 upregulation [206]. The link between cigarette smoking and the inflammatory microenvironment has been identified as a possible mechanism for cigarette smoking-induced oral carcinogenesis, tobacco extract (NNK) promoting miR-944 induction, activation of STAT3, and tumor malignancy through suppression of cytokine-inducible Src homology 2-containing (CISH) protein [201].

Dysregulation of miR-200 family miRNAs has been related to tobacco chewing/smoking, the cellular differentiation status of oral tumors, and upregulation of EMT-inducer genes in OSCC [202]. A potential role in the early development of smoking-related HNSCCs has been assigned to miR-1301, miR-101, miR-486, and miR-181b, miRNAs reported to be differentially expressed in cigarette-treated epithelial cell lines [203]. The involvement of alcohol in the early events of oral carcinogenesis was demonstrated in oral keratinocytes exposed to ethanol and acetaldehyde; thus, miR-3178, miR-934, miR-30a, and miR-3164 were upregulated, and the expression of miR-30a and miR-934 stimulated the induction of the anti-apoptotic gene BCL-2 in head and neck squamous cell carcinoma [88].

### 4.2. miRNAs Altered by Environmental Factors

TMPRSS2, a SARS-CoV-2 internalization protease, was identified as downregulated in HNSCC patients positive for SARS-CoV-2 infection, related to selective targeting of microRNAs, supporting the idea that tumor tissue from SARS-CoV-2 target organs is more resistant to SARS-CoV-2 infection [215]. The importance of miR-9 in human papillomavirus-associated with oral and oropharyngeal head and neck cancer was highlighted, with miR-9 being identified as the most important miRNA for HNSCC with HPV etiology and being upregulated in recurrent HNSCC [211]. In HPV16+ HNSCC tissues, a significant upregulation of miR-99a-3p and miR-4746-5p, and downregulation of miR-411-5p were observed; both miRNAs are involved in cancer progression through EMT-related pathways. The target genes are involved in cell growth and differentiation, MAPK, and FoxO signaling pathways [212]. Between the miR329/miR410 and the Wnt–β-catenin pathway, a strong relationship may be involved in oral carcinogenesis; dysregulation is associated with exposure to betel quid chewing [220]. miR-22 was downregulated via arecoline-induced c-Myc upregulation in arecoline-treated OSCC cells. Moreover, miR-22 inhibited proliferation, migration, and cell-cycle progression in OSCC cell lines [208]. In HPV (+) patients, miR-133a-3p is a target of smoking-induced modifications and was observed to be also significantly downregulated in OPSCC smokers and E6/E7 overexpressing HPV (−) cells that were treated with cigarette smoke extract [213].

HPV-positive oral carcinoma cells from smoking patients revealed a low activity of the Wnt/βCatenin pathway that can be related to the differential expression of miRNA let7e [214]. RNA deep sequencing was used to analyze the EBV-miR-BART1 involvement in the regulation of the cell’s metabolism-associated genes in nasopharyngeal carcinoma, demonstrating that EBV-miR-BART1, a virus-encoded miRNA, can be involved in cancer metabolism [221]. EBV-miR-BART7-3p was discovered to suppress SMAD7 in NPC stemness, leading to activated TGF-β signaling and drug resistance and cancer recurrence [217].

## 5. Conclusions

Most oral cancers are due to risk factors. Different altered miRNAs have been proven to play key roles in the initiation and progression of oral cancer due to their roles either as oncogenes or as tumor suppressors; they also have considerable potential to be excellent biomarkers and new therapeutic tools for this pathology. Furthermore, many studies have demonstrated a strong relation between altered miRNA expression and the main risk factors for the development of oral cancer, promoting early diagnosis and novel anticancer treatments to disturb the process of oral carcinogenesis. However, these studies require supplementation and validation in large cohorts of patients.

## Figures and Tables

**Table 1:** The main miRNAs related to risk factors in oral carcinogenesis.

Risk Factor	Tumor Type	miR	Targets	Effects/Clinical Significance	References
smoking	OSCC	miR-31-5p↑	SLC16A1	Cancer cell proliferation	[199]
	OSCC	miR-30a↓ miR-379↓	DNMT3B	Growth inhibition in oral cancer cells	[200]
	OSCC	miR-944↑	CISH STAT3	Maintaining a pro-carcinogenic microenvironment in oral cancer	[201]
	OSCC	miR-200a, miR-200b, miR-200c, miR-141 and miR-429, ↓	ZEB2-AS1 and ZEB2	No significant effect on treatment outcome	[202]
	HNSCC	miR-101-1, miR-181b-1, miR-486, and miR-1301↑		Increase of cell proliferation, metastasis, and decrease in survival	[203]
alcohol	HNSCC	miR-375↑		Decrease in survival	[204]
	OSCC	miR-34a↓	P53	Inhibition of tumor growth	[205]
		miR-30a↑miR-934↑	BCL-2	Increase in cellular proliferation	[88]
betel/tobacco chewing	OSCC	miR-155↑		Increase in cellular proliferation	[187]
	OSCC	miR-486-3p↓	DDR1	Growth inhibition and apoptosis induction	[206]
	OSCC	miR-30a↓ miR-379↓	DNMT3B	Growth inhibition in oral cancer cells	[200]
	OSCC	miR-29c-3p miR-146a-5p↑	SLC2A14 STAT 1, MX2, OASL	Cancer cells proliferation	[199]
	OSCC	miR329 and miR410↓	Wnt-7b	Proliferation and invasion of cells	[195]
Areca nut	OSCC	miR-23a↑	FANCG	Induction of cell proliferation	[207]
	OSCC	miR-22↓	OSM	Promote cell proliferation and cell-cycle progression	[208]
pan-masala chewing	OSCC	miR-21↑		Poor prognosis	[209]
HPV	TSCC/BOTSCC	miR-155↑ miR-193b↓ miR-185↑	CD8+ TIL	Decreased survival	[184]
	OSCC	miR-550a-3-5p↓	YAP CCL2	Larger tumor size and nodal metastasis	[210]
	HNSCC	miR-9↑		Proliferation and migration of the cells	[211]
	HNSCC	miR-99a-3p and miR-4746-5p↑ miR-411-5p↓	MAPK FoxO	Improvement of overall survival	[212]
	OPSCC	miR-133a-3p↓	EGFR and HuR	Promote cell proliferation	[213]
	OSCC	let-7e↑	βCatenin	Induction of stem-like traits in tobacco-related OSCCs	[214]
SARS-CoV-2	HNSCC	miR-193b-3p; miR-503-5p; miR-455-5p; miR-31-3p; miR-193b-5p; miR-2355-5p↑	TMPRSS2	Resistance to SARS-CoV-2 infection	[215]
EBV	NPC	EBV-miR-BART1↑	G6PD, SAT1, ASS1, PAST1, FUT1, SGPL1, DHRS3, PHGDH, GALNT1	Tumor metastasis	[216]
	NPC	miR-BART7-3p↑	SMAD7	Drug resistance and cancer recurrence	[217]

Abbreviations: TSCC/BOTSCC, tonsillar and base of tongue cancer; OSCC, oral squamous cell carcinoma; HNSCCs, head, and neck squamous cell carcinomas; OSM, oncostatin M; oropharyngeal squamous cell carcinoma (OPSCC); DNMT3B, DNA Methyltransferase 3 β; CISH, Cytokine Inducible SH2 Containing Protein; STAT3, Signal Transducer And Activator Of Transcription 3; ZEB2, Zinc Finger E-Box Binding Homeobox 2; ZEB2-AS1, ZEB2 Antisense RNA 1; BCL2, BCL2 Apoptosis Regulator; DDR1, Discoidin Domain Receptor Tyrosine Kinase 1; epidermal growth factor receptor (EGFR); SLC2A14, Solute Carrier Family 2 Member 14; MX2, MX Dynamin Like GTPase 2; OASL, 2′-5′-oligoadenylate synthase-like protein; YAP, Yes1 Associated Transcriptional Regulator HuR; FOXO1, Forkhead Box O1; TMPRSS2, Transmembrane Serine Protease 2; ASS1,Argininosuccinate Synthase 1; FUT1, Fucosyltransferase 1 (H Blood Group); SGPL1 (Sphingosine-1-Phosphate Lyase 1); DHRS3, dehydrogenase/reductase 3; PHGDH, Phosphoglycerate Dehydrogenase; RBP Hu-antigen R; GALNT1, Polypeptide N-Acetylgalactosaminyltransferase 1; NPC, nasopharyngeal carcinoma.

## References

[1] Trotta B.M., Pease C.S., Rasamny J.J., Raghavan P., Mukherjee S. (2011). Oral cavity and oropharyngeal squamous cell cancer: Key imaging findings for staging and treatment planning. Radiographics.

[2] Tseng H.H., Tseng Y.K., You J.J., Kang B.H., Wang T.H., Yang C.M., Chen H.C., Liou H.H., Liu P.F., Ger L.P. (2017). Next-generation Sequencing for microRNA Profiling: MicroRNA-21-3p Promotes Oral Cancer Metastasis. Anticancer. Res..

[3] Chang T.S., Chang C.M., Ho H.C., Su Y.C., Chen L.F., Chou P., Lee C.C. (2013). Impact of young age on the prognosis for oral cancer: A population-based study in Taiwan. PLoS ONE.

[4] Ferlay J., Colombet M., Soerjomataram I., Mathers C., Parkin D.M., Piñeros M., Znaor A., Bray F. (2019). Estimating the global cancer incidence and mortality in 2018: GLOBOCAN sources and methods. Int. J. Cancer.

[5] Van der Waal I. (2013). Are we able to reduce the mortality and morbidity of oral cancer; some considerations. Med. Oral Patol. Oral Y Cir. Bucal.

[6] Ong T.K., Murphy C., Smith A.B., Kanatas A.N., Mitchell D.A. (2017). Survival after surgery for oral cancer: A 30-year experience. Br. J. Oral Maxillofac. Surg..

[7] Tsai W.C., Kung P.T., Wang S.T., Huang K.H., Liu S.A. (2015). Beneficial impact of multidisciplinary team management on the survival in different stages of oral cavity cancer patients: Results of a nationwide cohort study in Taiwan. Oral Oncol..

[8] Wyss A., Hashibe M., Chuang S.C., Lee Y.C., Zhang Z.F., Yu G.P., Winn D.M., Wei Q., Talamini R., Szeszenia-Dabrowska N. (2013). Cigarette, cigar, and pipe smoking and the risk of head and neck cancers: Pooled analysis in the International Head and Neck Cancer Epidemiology Consortium. Am. J. Epidemiol..

[9] Inchingolo F., Santacroce L., Ballini A., Topi S., Dipalma G., Haxhirexha K., Bottalico L., Charitos I.A. (2020). Oral Cancer: A Historical Review. Int. J. Environ. Res. Public Health.

[10] Warnakulasuriya S. (2009). Global epidemiology of oral and oropharyngeal cancer. Oral Oncol..

[11] Hsiao J.R., Chang C.C., Lee W.T., Huang C.C., Ou C.Y., Tsai S.T., Chen K.C., Huang J.S., Wong T.Y., Lai Y.H. (2018). The interplay between oral microbiome, lifestyle factors and genetic polymorphisms in the risk of oral squamous cell carcinoma. Carcinogenesis.

[12] Chang Y.A., Weng S.L., Yang S.F., Chou C.H., Huang W.C., Tu S.J., Chang T.H., Huang C.N., Jong Y.J., Huang H.D. (2018). A Three-MicroRNA Signature as a Potential Biomarker for the Early Detection of Oral Cancer. Int. J. Mol. Sci..

[13] Hung K.F., Liu C.J., Chiu P.C., Lin J.S., Chang K.W., Shih W.Y., Kao S.Y., Tu H.F. (2016). MicroRNA-31 upregulation predicts increased risk of progression of oral potentially malignant disorder. Oral Oncol..

[14] Prete R.D., Ronga L., Addati G., Magrone R., Abbasciano A., Carlo D.D., Santacroce L. (2019). A Retrospective Study about the Impact of Switching from Nested PCR to Multiplex Real-Time PCR on the Distribution of the Human Papillomavirus (HPV) Genotypes. Medicina.

[15] Mehanna H., Beech T., Nicholson T., El-Hariry I., McConkey C., Paleri V., Roberts S. (2013). Prevalence of human papillomavirus in oropharyngeal and nonoropharyngeal head and neck cancer—Systematic review and meta-analysis of trends by time and region. Head Neck.

[16] Tatullo M., Gentile S., Paduano F., Santacroce L., Marrelli M. (2016). Crosstalk between oral and general health status in e-smokers. Medicine.

[17] Irimie A.I., Braicu C., Cojocneanu R., Magdo L., Onaciu A., Ciocan C., Mehterov N., Dudea D., Buduru S., Berindan-Neagoe I. (2018). Differential Effect of Smoking on Gene Expression in Head and Neck Cancer Patients. Int J. Environ. Res. Public Health.

[18] Hashibe M., Brennan P., Chuang S.C., Boccia S., Castellsague X., Chen C., Curado M.P., Dal Maso L., Daudt A.W., Fabianova E. (2009). Interaction between tobacco and alcohol use and the risk of head and neck cancer: Pooled analysis in the International Head and Neck Cancer Epidemiology Consortium. Cancer Epidemiol. Biomark. Prev..

[19] Zandberg D.P., Liu S., Goloubeva O., Ord R., Strome S.E., Suntharalingam M., Taylor R., Morales R.E., Wolf J.S., Zimrin A. (2016). Oropharyngeal cancer as a driver of racial outcome disparities in squamous cell carcinoma of the head and neck: 10-year experience at the University of Maryland Greenebaum Cancer Center. Head Neck.

[20] Chang E.T., Liu Z., Hildesheim A., Liu Q., Cai Y., Zhang Z., Chen G., Xie S.H., Cao S.M., Shao J.Y. (2017). Active and Passive Smoking and Risk of Nasopharyngeal Carcinoma: A Population-Based Case-Control Study in Southern China. Am. J. Epidemiol..

[21] Whiteman D.C., Wilson L.F. (2016). The fractions of cancer attributable to modifiable factors: A global review. Cancer Epidemiol..

[22] Bray F., Ferlay J., Soerjomataram I., Siegel R.L., Torre L.A., Jemal A. (2018). Global cancer statistics 2018: GLOBOCAN estimates of incidence and mortality worldwide for 36 cancers in 185 countries. CA Cancer J. Clin..

[23] de Martel C., Plummer M., Vignat J., Franceschi S. (2017). Worldwide burden of cancer attributable to HPV by site, country and HPV type. Int. J. Cancer.

[24] Martin-Gomez L., Giuliano A.R., Fulp W.J., Caudell J., Echevarria M., Sirak B., Abrahamsen M., Isaacs-Soriano K.A., Hernandez-Prera J.C., Wenig B.M. (2019). Human Papillomavirus Genotype Detection in Oral Gargle Samples Among Men With Newly Diagnosed Oropharyngeal Squamous Cell Carcinoma. JAMA Otolaryngol. Head Neck Surg..

[25] Shaw R.J., Liloglou T., Rogers S.N., Brown J.S., Vaughan E.D., Lowe D., Field J.K., Risk J.M. (2006). Promoter methylation of P16, RARbeta, E-cadherin, cyclin A1 and cytoglobin in oral cancer: Quantitative evaluation using pyrosequencing. Br. J. Cancer.

[26] Kulkarni V., Saranath D. (2004). Concurrent hypermethylation of multiple regulatory genes in chewing tobacco associated oral squamous cell carcinomas and adjacent normal tissues. Oral Oncol..

[27] D’Souza W., Saranath D. (2015). Clinical implications of epigenetic regulation in oral cancer. Oral Oncol..

[28] Bai Z.-T., Bai B., Zhu J., Di C.-X., Li X., Zhou W.-C. (2018). Epigenetic actions of environmental factors and promising drugs for cancer therapy. Oncol. Lett..

[29] Arif K.M.T., Elliott E.K., Haupt L.M., Griffiths L.R. (2020). Regulatory Mechanisms of Epigenetic miRNA Relationships in Human Cancer and Potential as Therapeutic Targets. Cancers.

[30] Irimie A.I., Ciocan C., Gulei D., Mehterov N., Atanasov A.G., Dudea D., Berindan-Neagoe I. (2018). Current Insights into Oral Cancer Epigenetics. Int. J. Mol. Sci..

[31] Jin B., Li Y., Robertson K.D. (2011). DNA methylation: Superior or subordinate in the epigenetic hierarchy?. Genes Cancer.

[32] Koch A., Joosten S.C., Feng Z., de Ruijter T.C., Draht M.X., Melotte V., Smits K.M., Veeck J., Herman J.G., Van Neste L. (2018). Analysis of DNA methylation in cancer: Location revisited. Nat. Rev. Clin. Oncol..

[33] Loaeza-Loaeza J., Beltran A.S., Hernández-Sotelo D. (2020). DNMTs and Impact of CpG Content, Transcription Factors, Consensus Motifs, lncRNAs, and Histone Marks on DNA Methylation. Genes.

[34] Horii T., Hatada I. (2016). Regulation of CpG methylation by Dnmt and Tet in pluripotent stem cells. J. Reprod. Dev..

[35] Saatci C., Caglayan A.O., Ozkul Y., Tahiri S., Turhan A.B., Dundar M. (2009). Detection of p16 promotor hypermethylation in “Maras powder” and tobacco users. Cancer Epidemiol..

[36] Takeshima M., Saitoh M., Kusano K., Nagayasu H., Kurashige Y., Malsantha M., Arakawa T., Takuma T., Chiba I., Kaku T. (2008). High frequency of hypermethylation of p14, p15 and p16 in oral pre-cancerous lesions associated with betel-quid chewing in Sri Lanka. J. Oral Pathol. Med..

[37] Liu C., Marioni R.E., Hedman Å K., Pfeiffer L., Tsai P.C., Reynolds L.M., Just A.C., Duan Q., Boer C.G., Tanaka T. (2018). A DNA methylation biomarker of alcohol consumption. Mol. Psychiatry.

[38] Kato K., Hara A., Kuno T., Mori H., Yamashita T., Toida M., Shibata T. (2006). Aberrant promoter hypermethylation of p16 and MGMT genes in oral squamous cell carcinomas and the surrounding normal mucosa. J. Cancer Res. Clin. Oncol..

[39] Hema K.N., Smitha T., Sheethal H.S., Mirnalini S.A. (2017). Epigenetics in oral squamous cell carcinoma. J. Oral Maxillofac. Pathol..

[40] Goodchild M., Nargis N., Tursan d’Espaignet E. (2018). Global economic cost of smoking-attributable diseases. Tob. Control..

[41] Jiang X., Wu J., Wang J., Huang R. (2019). Tobacco and oral squamous cell carcinoma: A review of carcinogenic pathways. Tob. Induc. Dis..

[42] Getz K.R., Rozek L.S., Peterson L.A., Bellile E.L., Taylor J.M.G., Wolf G.T., Mondul A.M. (2017). Family history of cancer and head and neck cancer survival. Laryngoscope.

[43] Dhull A.K., Atri R., Dhankhar R., Chauhan A.K., Kaushal V. (2018). Major Risk Factors in Head and Neck Cancer: A Retrospective Analysis of 12-Year Experiences. World J. Oncol..

[44] Maasland D.H., van den Brandt P.A., Kremer B., Goldbohm R.A., Schouten L.J. (2014). Alcohol consumption, cigarette smoking and the risk of subtypes of head-neck cancer: Results from the Netherlands Cohort Study. BMC Cancer.

[45] Chrcanovic B.R., Albrektsson T., Wennerberg A. (2015). Smoking and dental implants: A systematic review and meta-analysis. J. Dent..

[46] Ramôa C.P., Eissenberg T., Sahingur S.E. (2017). Increasing popularity of waterpipe tobacco smoking and electronic cigarette use: Implications for oral healthcare. J. Periodontal Res..

[47] Pemberton M.N. (2018). Oral cancer and tobacco: Developments in harm reduction. Br. Dent. J..

[48] Maki J. (2015). The incentives created by a harm reduction approach to smoking cessation: Snus and smoking in Sweden and Finland. Int. J. Drug Policy.

[49] Kumar M., Nanavati R., Modi T.G., Dobariya C. (2016). Oral cancer: Etiology and risk factors: A review. J. Cancer Res. Ther..

[50] Lubin J.H., Purdue M., Kelsey K., Zhang Z.F., Winn D., Wei Q., Talamini R., Szeszenia-Dabrowska N., Sturgis E.M., Smith E. (2009). Total exposure and exposure rate effects for alcohol and smoking and risk of head and neck cancer: A pooled analysis of case-control studies. Am. J. Epidemiol..

[51] Farsalinos K.E., Yannovits N., Sarri T., Voudris V., Poulas K. (2018). Nicotine Delivery to the Aerosol of a Heat-Not-Burn Tobacco Product: Comparison With a Tobacco Cigarette and E-Cigarettes. Nicotine Tob. Res..

[52] Li X., Luo Y., Jiang X., Zhang H., Zhu F., Hu S., Hou H., Hu Q., Pang Y. (2019). Chemical Analysis and Simulated Pyrolysis of Tobacco Heating System 2.2 Compared to Conventional Cigarettes. Nicotine Tob. Res..

[53] Zanetti F., Titz B., Sewer A., Lo Sasso G., Scotti E., Schlage W.K., Mathis C., Leroy P., Majeed S., Torres L.O. (2017). Comparative systems toxicology analysis of cigarette smoke and aerosol from a candidate modified risk tobacco product in organotypic human gingival epithelial cultures: A 3-day repeated exposure study. Food Chem. Toxicol..

[54] Zanetti F., Sewer A., Mathis C., Iskandar A.R., Kostadinova R., Schlage W.K., Leroy P., Majeed S., Guedj E., Trivedi K. (2016). Systems Toxicology Assessment of the Biological Impact of a Candidate Modified Risk Tobacco Product on Human Organotypic Oral Epithelial Cultures. Chem. Res. Toxicol..

[55] Zanetti F., Sewer A., Scotti E., Titz B., Schlage W.K., Leroy P., Kondylis A., Vuillaume G., Iskandar A.R., Guedj E. (2018). Assessment of the impact of aerosol from a potential modified risk tobacco product compared with cigarette smoke on human organotypic oral epithelial cultures under different exposure regimens. Food Chem. Toxicol..

[56] Clarke E., Thompson K., Weaver S., Thompson J., O’Connell G. (2019). Snus: A compelling harm reduction alternative to cigarettes. Harm Reduct. J..

[57] Talukdar F.R., Ghosh S.K., Laskar R.S., Mondal R. (2013). Epigenetic, Genetic and Environmental Interactions in Esophageal Squamous Cell Carcinoma from Northeast India. PLoS ONE.

[58] Yuan J.M., Murphy S.E., Stepanov I., Wang R., Carmella S.G., Nelson H.H., Hatsukami D., Hecht S.S. (2016). 2-Phenethyl Isothiocyanate, Glutathione S-transferase M1 and T1 Polymorphisms, and Detoxification of Volatile Organic Carcinogens and Toxicants in Tobacco Smoke. Cancer Prev. Res..

[59] Patel K., Bhat F.A., Patil S., Routray S., Mohanty N., Nair B., Sidransky D., Ganesh M.S., Ray J.G., Gowda H. (2021). Whole-Exome Sequencing Analysis of Oral Squamous Cell Carcinoma Delineated by Tobacco Usage Habits. Front. Oncol..

[60] Sun Y.W., Chen K.M., Imamura Kawasawa Y., Salzberg A.C., Cooper T.K., Caruso C., Aliaga C., Zhu J., Gowda K., Amin S. (2017). Hypomethylated Fgf3 is a potential biomarker for early detection of oral cancer in mice treated with the tobacco carcinogen dibenzo[def,p]chrysene. PLoS ONE.

[61] Ghantous Y., Schussel J.L., Brait M. (2018). Tobacco and alcohol-induced epigenetic changes in oral carcinoma. Curr. Opin. Oncol..

[62] Chang H.W., Ling G.S., Wei W.I., Yuen A.P. (2004). Smoking and drinking can induce p15 methylation in the upper aerodigestive tract of healthy individuals and patients with head and neck squamous cell carcinoma. Cancer.

[63] Breitling L.P., Yang R., Korn B., Burwinkel B., Brenner H. (2011). Tobacco-smoking-related differential DNA methylation: 27K discovery and replication. Am. J. Hum. Genet..

[64] Furniss C.S., Marsit C.J., Houseman E.A., Eddy K., Kelsey K.T. (2008). Line region hypomethylation is associated with lifestyle and differs by human papillomavirus status in head and neck squamous cell carcinomas. Cancer Epidemiol. Biomark. Prev..

[65] Supic G., Kozomara R., Jovic N., Zeljic K., Magic Z. (2011). Prognostic significance of tumor-related genes hypermethylation detected in cancer-free surgical margins of oral squamous cell carcinomas. Oral Oncol..

[66] Khan Z., Khan S., Christianson L., Rehman S., Ekwunife O., Samkange-Zeeb F. (2017). Smokeless Tobacco and Oral Potentially Malignant Disorders in South Asia: A Systematic Review and Meta-analysis. Nicotine Tob. Res..

[67] Islam S., Muthumala M., Matsuoka H., Uehara O., Kuramitsu Y., Chiba I., Abiko Y. (2019). How Each Component of Betel Quid Is Involved in Oral Carcinogenesis: Mutual Interactions and Synergistic Effects with Other Carcinogens—A Review Article. Curr. Oncol. Rep..

[68] Sharan R.N., Mehrotra R., Choudhury Y., Asotra K. (2012). Association of betel nut with carcinogenesis: Revisit with a clinical perspective. PLoS ONE.

[69] Athukorala I.A., Tilakaratne W.M., Jayasinghe R.D. (2021). Areca Nut Chewing: Initiation, Addiction, and Harmful Effects Emphasizing the Barriers and Importance of Cessation. J. Addict..

[70] Anand R., Dhingra C., Prasad S., Menon I. (2014). Betel nut chewing and its deleterious effects on oral cavity. J. Cancer Res. Ther..

[71] Lai Z.L., Tsou Y.A., Fan S.R., Tsai M.H., Chen H.L., Chang N.W., Cheng J.C., Chen C.M. (2014). Methylation-associated gene silencing of RARB in areca carcinogens induced mouse oral squamous cell carcinoma. BioMed Res. Int..

[72] Chang M.C., Chan C.P., Chen Y.J., Hsien H.C., Chang Y.C., Yeung S.Y., Jeng P.Y., Cheng R.H., Hahn L.J., Jeng J.H. (2016). Areca nut components stimulate ADAM17, IL-1α, PGE2 and 8-isoprostane production in oral keratinocyte: Role of reactive oxygen species, EGF and JAK signaling. Oncotarget.

[73] GBD 2016 Alcohol Collaborators (2018). Alcohol use and burden for 195 countries and territories, 1990–2016: A systematic analysis for the Global Burden of Disease Study 2016. Lancet.

[74] Wang T.H., Hsia S.M., Shih Y.H., Shieh T.M. (2017). Association of Smoking, Alcohol Use, and Betel Quid Chewing with Epigenetic Aberrations in Cancers. Int. J. Mol. Sci..

[75] Rehm J., Mathers C., Popova S., Thavorncharoensap M., Teerawattananon Y., Patra J. (2009). Global burden of disease and injury and economic cost attributable to alcohol use and alcohol-use disorders. Lancet.

[76] Marziliano A., Teckie S., Diefenbach M.A. (2020). Alcohol-related head and neck cancer: Summary of the literature. Head Neck.

[77] Seitz H.K., Stickel F. (2010). Acetaldehyde as an underestimated risk factor for cancer development: Role of genetics in ethanol metabolism. Genes Nutr..

[78] Chang C.P., Siwakoti B., Sapkota A., Gautam D.K., Lee Y.A., Monroe M., Hashibe M. (2020). Tobacco smoking, chewing habits, alcohol drinking and the risk of head and neck cancer in Nepal. Int. J. Cancer.

[79] Wang R., Li B., Jiang Y., Guan Y., Wang G., Zhao G. (2019). Smoking cessation mutually facilitates alcohol drinking cessation among tobacco and alcohol co-users: A cross-sectional study in a rural area of Shanghai, China. Tob. Induc. Dis..

[80] Kawakita D., Matsuo K. (2017). Alcohol and head and neck cancer. Cancer Metastasis Rev..

[81] Quertemont E. (2004). Genetic polymorphism in ethanol metabolism: Acetaldehyde contribution to alcohol abuse and alcoholism. Mol. Psychiatry.

[82] Tang K., Li Y., Zhang Z., Gu Y., Xiong Y., Feng G., He L., Qin S. (2010). The PstI/RsaI and DraI polymorphisms of CYP2E1 and head and neck cancer risk: A meta-analysis based on 21 case-control studies. BMC Cancer.

[83] Ramani V.K., Vinod G.D., Benny N., Naik R. (2021). Characteristics of tobacco consumption among cancer patients at a tertiary cancer hospital in South India-A cross-sectional study. Tob. Use Insights.

[84] Hashibe M., Brennan P., Benhamou S., Castellsague X., Chen C., Curado M.P., Dal Maso L., Daudt A.W., Fabianova E., Fernandez L. (2007). Alcohol drinking in never users of tobacco, cigarette smoking in never drinkers, and the risk of head and neck cancer: Pooled analysis in the International Head and Neck Cancer Epidemiology Consortium. J. Natl. Cancer Inst..

[85] Silva P., Latruffe N., Gaetano G. (2020). Wine Consumption and Oral Cavity Cancer: Friend or Foe, Two Faces of Janus. Molecules.

[86] Theruvathu J.A., Jaruga P., Nath R.G., Dizdaroglu M., Brooks P.J. (2005). Polyamines stimulate the formation of mutagenic 1,N2-propanodeoxyguanosine adducts from acetaldehyde. Nucleic Acids Res..

[87] Yu V., Singh P., Rahimy E., Zheng H., Kuo S.Z., Kim E., Wang-Rodriguez J., Ongkeko W.M. (2016). RNA-seq analysis identifies key long non-coding RNAs connected to the pathogenesis of alcohol-associated head and neck squamous cell carcinoma. Oncol. Lett..

[88] Saad M.A., Kuo S.Z., Rahimy E., Zou A.E., Korrapati A., Rahimy M., Kim E., Zheng H., Yu M.A., Wang-Rodriguez J. (2015). Alcohol-dysregulated miR-30a and miR-934 in head and neck squamous cell carcinoma. Mol. Cancer.

[89] Gupta B., Bray F., Kumar N., Johnson N.W. (2017). Associations between oral hygiene habits, diet, tobacco and alcohol and risk of oral cancer: A case-control study from India. Cancer Epidemiol..

[90] Bravi F., Bosetti C., Filomeno M., Levi F., Garavello W., Galimberti S., Negri E., La Vecchia C. (2013). Foods, nutrients and the risk of oral and pharyngeal cancer. Br. J. Cancer.

[91] Chuang S.C., Jenab M., Heck J.E., Bosetti C., Talamini R., Matsuo K., Castellsague X., Franceschi S., Herrero R., Winn D.M. (2012). Diet and the risk of head and neck cancer: A pooled analysis in the INHANCE consortium. Cancer Causes Control..

[92] Pelucchi C., Bosetti C., Negri E., Lipworth L., La Vecchia C. (2011). Olive oil and cancer risk: An update of epidemiological findings through 2010. Curr. Pharm. Des..

[93] Nosrati N., Bakovic M., Paliyath G. (2017). Molecular Mechanisms and Pathways as Targets for Cancer Prevention and Progression with Dietary Compounds. Int. J. Mol. Sci..

[94] Toporcov T.N., Tavares G.E., Rotundo L.D., Vaccarezza G.F., Biazevic M.G., Brasileiro R.S., de Carvalho M.B., Michaluart P., Kowalski L.P., Antunes J.L. (2012). Do tobacco and alcohol modify protective effects of diet on oral carcinogenesis?. Nutr. Cancer.

[95] Rossi M., Garavello W., Talamini R., Negri E., Bosetti C., Dal Maso L., Lagiou P., Tavani A., Polesel J., Barzan L. (2007). Flavonoids and the risk of oral and pharyngeal cancer: A case-control study from Italy. Cancer Epidemiol. Biomark. Prev..

[96] Irimie A.I., Braicu C., Zanoaga O., Pileczki V., Gherman C., Berindan-Neagoe I., Campian R.S. (2015). Epigallocatechin-3-gallate suppresses cell proliferation and promotes apoptosis and autophagy in oral cancer SSC-4 cells. OncoTargets Ther..

[97] Bauman J.E., Zang Y., Sen M., Li C., Wang L., Egner P.A., Fahey J.W., Normolle D.P., Grandis J.R., Kensler T.W. (2016). Prevention of Carcinogen-Induced Oral Cancer by Sulforaphane. Cancer Prev. Res..

[98] Galvão De Podestá O.P., Peres S.V., Salaroli L.B., Cattafesta M., De Podestá J.R.V., von Zeidler S.L.V., de Oliveira J.C., Kowalski L.P., Ikeda M.K., Brennan P. (2019). Consumption of minimally processed foods as protective factors in the genesis of squamous cell carcinoma of the head and neck in Brazil. PLoS ONE.

[99] Green J.M., Ciancio M.J., Goral J., Pytynia M., Pitstick L., Meyer A., Nguyen A., Lee K., Barakat A., Jham B.C. (2021). Dietary fat and male sex increase histopathological changes in a mouse model of oral cancer. Oral Dis..

[100] Rodríguez-Molinero J., Migueláñez-Medrán B.D.C., Puente-Gutiérrez C., Delgado-Somolinos E., Martín Carreras-Presas C., Fernández-Farhall J., López-Sánchez A.F. (2021). Association between Oral Cancer and Diet: An Update. Nutrients.

[101] Peng J., Hu Q., Chen X., Wang C., Zhang J., Ren X., Wang Y., Tao X., Li H., Song M. (2021). Diet-induced obesity accelerates oral carcinogenesis by recruitment and functional enhancement of myeloid-derived suppressor cells. Cell Death Dis..

[102] Gandini S., Negri E., Boffetta P., La Vecchia C., Boyle P. (2012). Mouthwash and oral cancer risk quantitative meta-analysis of epidemiologic studies. Ann. Agric. Environ. Med..

[103] Ustrell-Borràs M., Traboulsi-Garet B., Gay-Escoda C. (2020). Alcohol-based mouthwash as a risk factor of oral cancer: A systematic review. Med. Oral Patol. Oral Y Cir. Bucal.

[104] Aceves Argemí R., González Navarro B., Ochoa García-Seisdedos P., Estrugo Devesa A., López-López J. (2020). Mouthwash With Alcohol and Oral Carcinogenesis: Systematic Review and Meta-analysis. J. Evid. -Based Dent. Pract..

[105] Tumban E. (2019). A Current Update on Human Papillomavirus-Associated Head and Neck Cancers. Viruses.

[106] Li H., Torabi S.J., Yarbrough W.G., Mehra S., Osborn H.A., Judson B. (2018). Association of Human Papillomavirus Status at Head and Neck Carcinoma Subsites With Overall Survival. JAMA Otolaryngol. Head Neck Surg..

[107] Gillison M.L., Chaturvedi A.K., Anderson W.F., Fakhry C. (2015). Epidemiology of Human Papillomavirus-Positive Head and Neck Squamous Cell Carcinoma. J. Clin. Oncol..

[108] Devaraja K., Aggarwal S., Verma S.S., Gupta S.C. (2020). Clinico-pathological peculiarities of human papilloma virus driven head and neck squamous cell carcinoma: A comprehensive update. Life Sci..

[109] Lin N.C., Hsu J.T., Tsai K.Y. (2020). Difference between Female and Male Patients with Oral Squamous Cell Carcinoma: A Single-Center Retrospective Study in Taiwan. Int. J. Environ. Res. Public Health.

[110] Auguste A., Deloumeaux J., Joachim C., Gaete S., Michineau L., Herrmann-Storck C., Duflo S., Luce D. (2020). Joint effect of tobacco, alcohol, and oral HPV infection on head and neck cancer risk in the French West Indies. Cancer Med..

[111] Nair S., Pillai M.R. (2005). Human papillomavirus and disease mechanisms: Relevance to oral and cervical cancers. Oral Dis..

[112] Fakhry C., Westra W.H., Li S., Cmelak A., Ridge J.A., Pinto H., Forastiere A., Gillison M.L. (2008). Improved survival of patients with human papillomavirus-positive head and neck squamous cell carcinoma in a prospective clinical trial. J. Natl. Cancer Inst..

[113] Chaturvedi A.K., Engels E.A., Pfeiffer R.M., Hernandez B.Y., Xiao W., Kim E., Jiang B., Goodman M.T., Sibug-Saber M., Cozen W. (2011). Human papillomavirus and rising oropharyngeal cancer incidence in the United States. J. Clin. Oncol..

[114] Robayo D.A.G., Erira H.A.T., Jaimes F.O.G., Torres A.M., Galindo A.I.C. (2019). Oropharyngeal Squamous Cell Carcinoma: Human Papilloma Virus Coinfection with *Streptococcus anginosus*. Braz. Dent. J..

[115] Di Domenico M., Giovane G., Kouidhi S., Iorio R., Romano M., De Francesco F., Feola A., Siciliano C., Califano L., Giordano A. (2018). HPV epigenetic mechanisms related to Oropharyngeal and Cervix cancers. Cancer Biol. Ther..

[116] Visalli G., Currò M., Facciolà A., Riso R., Mondello P., Laganà P., Di Pietro A., Picerno I., Spataro P. (2016). Prevalence of human papillomavirus in saliva of women with HPV genital lesions. Infect. Agents Cancer.

[117] Yen C.Y., Lu M.C., Tzeng C.C., Huang J.Y., Chang H.W., Chen R.S., Liu S.Y., Liu S.T., Shieh B., Li C. (2009). Detection of EBV infection and gene expression in oral cancer from patients in Taiwan by microarray analysis. J. Biomed. Biotechnol..

[118] Ram H., Sarkar J., Kumar H., Konwar R., Bhatt M.L., Mohammad S. (2011). Oral cancer: Risk factors and molecular pathogenesis. J. Maxillofac. Oral Surg..

[119] De Lima M.A.P., Teodoro I.P.P., Galiza L.E., Filho P., Marques F.M., Pinheiro Junior R.F.F., Macedo G.E.C., Facundo H.T., da Silva C.G.L., Lima M.V.A. (2019). Association between Epstein-Barr Virus and Oral Carcinoma: A Systematic Review with Meta-Analysis. Crit. Rev. Oncog..

[120] Kis A., Fehér E., Gáll T., Tar I., Boda R., Tóth E.D., Méhes G., Gergely L., Szarka K. (2009). Epstein-Barr virus prevalence in oral squamous cell cancer and in potentially malignant oral disorders in an eastern Hungarian population. Eur. J. Oral Sci..

[121] Eliopoulos A.G., Young L.S. (2001). LMP1 structure and signal transduction. Semin. Cancer Biol..

[122] Blandino G. (2020). Cancer at the time of the COVID-19 hurricane. J. Exp. Clin. Cancer Res..

[123] Silvestris N., Apolone G., Botti G., Ciliberto G., Costantini M., De Paoli P., Franceschi S., Opocher G., Paradiso A., Pronzato P. (2020). A moonshot approach toward the management of cancer patients in the COVID-19 time: What have we learned and what could the Italian network of cancer centers (Alliance Against Cancer, ACC) do after the pandemic wave?. J. Exp. Clin. Cancer Res..

[124] Mariz B., Brandão T.B., Ribeiro A.C.P., Lopes M.A., Santos-Silva A.R. (2020). New Insights for the Pathogenesis of COVID-19-Related Dysgeusia. J. Dent. Res..

[125] Brandão T.B., Gueiros L.A., Melo T.S., Prado-Ribeiro A.C., Nesrallah A., Prado G.V.B., Santos-Silva A.R., Migliorati C.A. (2021). Oral lesions in patients with SARS-CoV-2 infection: Could the oral cavity be a target organ?. Oral Surg. Oral Med. Oral Pathol. Oral Radiol..

[126] Engku Nasrullah Satiman E.A.F., Ahmad H., Ramzi A.B., Abdul Wahab R., Kaderi M.A., Wan Harun W.H.A., Dashper S., McCullough M., Arzmi M.H. (2020). The role of *Candida albicans* candidalysin ECE1 gene in oral carcinogenesis. J. Oral Pathol. Med..

[127] Alnuaimi A.D., Wiesenfeld D., O’Brien-Simpson N.M., Reynolds E.C., McCullough M.J. (2015). Oral *Candida* colonization in oral cancer patients and its relationship with traditional risk factors of oral cancer: A matched case-control study. Oral Oncol..

[128] Inaba H., Sugita H., Kuboniwa M., Iwai S., Hamada M., Noda T., Morisaki I., Lamont R.J., Amano A. (2014). *Porphyromonas gingivalis* promotes invasion of oral squamous cell carcinoma through induction of proMMP9 and its activation. Cell. Microbiol..

[129] Meurman J.H., Bascones-Martinez A. (2011). Are oral and dental diseases linked to cancer?. Oral Dis..

[130] Galvão-Moreira L.V., da Cruz M.C. (2016). Oral microbiome, periodontitis and risk of head and neck cancer. Oral Oncol..

[131] Groeger S., Domann E., Gonzales J.R., Chakraborty T., Meyle J. (2011). B7-H1 and B7-DC receptors of oral squamous carcinoma cells are upregulated by *Porphyromonas gingivalis*. Immunobiology.

[132] Rai A.K., Panda M., Das A.K., Rahman T., Das R., Das K., Sarma A., Kataki A.C., Chattopadhyay I. (2021). Dysbiosis of salivary microbiome and cytokines influence oral squamous cell carcinoma through inflammation. Arch. Microbiol..

[133] Alnuaimi A.D., Ramdzan A.N., Wiesenfeld D., O’Brien-Simpson N.M., Kolev S.D., Reynolds E.C., McCullough M.J. (2016). Candida virulence and ethanol-derived acetaldehyde production in oral cancer and non-cancer subjects. Oral Dis..

[134] Ndiaye C., Mena M., Alemany L., Arbyn M., Castellsagué X., Laporte L., Bosch F.X., de Sanjosé S., Trottier H. (2014). HPV DNA, E6/E7 mRNA, and p16INK4a detection in head and neck cancers: A systematic review and meta-analysis. Lancet Oncol..

[135] Auluck A., Walker B.B., Hislop G., Lear S.A., Schuurman N., Rosin M. (2016). Socio-economic deprivation: A significant determinant affecting stage of oral cancer diagnosis and survival. BMC Cancer.

[136] Hung L.C., Kung P.T., Lung C.H., Tsai M.H., Liu S.A., Chiu L.T., Huang K.H., Tsai W.C. (2020). Assessment of the Risk of Oral Cancer Incidence in A High-Risk Population and Establishment of A Predictive Model for Oral Cancer Incidence Using A Population-Based Cohort in Taiwan. Int. J. Environ. Res. Public Health.

[137] Paget-Bailly S., Cyr D., Luce D. (2012). Occupational exposures to asbestos, polycyclic aromatic hydrocarbons and solvents, and cancers of the oral cavity and pharynx: A quantitative literature review. Int. Arch. Occup. Environ. Health.

[138] Smailyte G. (2012). Cancer incidence among workers exposed to softwood dust in Lithuania. Occup. Environ. Med..

[139] Hashim D., Boffetta P. (2014). Occupational and environmental exposures and cancers in developing countries. Ann. Glob. Health.

[140] Adrien J., Bertolus C., Gambotti L., Mallet A., Baujat B. (2014). Why are head and neck squamous cell carcinoma diagnosed so late? Influence of health care disparities and socio-economic factors. Oral Oncol..

[141] Dholam K.P., Chouksey G.C. (2016). Squamous cell carcinoma of the oral cavity and oropharynx in patients aged 18–45 years: A case-control study to evaluate the risk factors with emphasis on stress, diet, oral hygiene, and family history. Indian J. Cancer.

[142] Hashim D., Sartori S., Brennan P., Curado M.P., Wünsch-Filho V., Divaris K., Olshan A.F., Zevallos J.P., Winn D.M., Franceschi S. (2016). The role of oral hygiene in head and neck cancer: Results from International Head and Neck Cancer Epidemiology (INHANCE) consortium. Ann. Oncol..

[143] Kawakita D., Lee Y.A., Li Q., Chen Y., Chen C.J., Hsu W.L., Lou P.J., Zhu C., Pan J., Shen H. (2017). Impact of oral hygiene on head and neck cancer risk in a Chinese population. Head Neck.

[144] Mathur R., Singhavi H.R., Malik A., Nair S., Chaturvedi P. (2019). Role of Poor Oral Hygiene in Causation of Oral Cancer-a Review of Literature. Indian J. Surg. Oncol..

[145] Warnakulasuriya S. (2009). Causes of oral cancer—An appraisal of controversies. Br. Dent. J..

[146] Garavello W., Foschi R., Talamini R., La Vecchia C., Rossi M., Dal Maso L., Tavani A., Levi F., Barzan L., Ramazzotti V. (2008). Family history and the risk of oral and pharyngeal cancer. Int. J. Cancer.

[147] Radoï L., Paget-Bailly S., Guida F., Cyr D., Menvielle G., Schmaus A., Carton M., Cénée S., Sanchez M., Guizard A.V. (2013). Family history of cancer, personal history of medical conditions and risk of oral cavity cancer in France: The ICARE study. BMC Cancer.

[148] Brown L.M., Gridley G., Diehl S.R., Winn D.M., Harty L.C., Otero E.B., Fraumeni J.F., Hayes R.B. (2001). Family cancer history and susceptibility to oral carcinoma in Puerto Rico. Cancer.

[149] Goldgar D.E., Easton D.F., Cannon-Albright L.A., Skolnick M.H. (1994). Systematic population-based assessment of cancer risk in first-degree relatives of cancer probands. J. Natl. Cancer Inst..

[150] Chen S., Lin Z., Chen J., Yang A., Zhang Q., Xie C., Zhang X., Yang Z., Chen W., Song M. (2020). Older age is a risk factor associated with poor prognosis of patients with squamous cell carcinoma of the oral cavity. Eur. Arch. Oto-Rhino-Laryngol..

[151] Lin W.J., Jiang R.S., Wu S.H., Chen F.J., Liu S.A. (2011). Smoking, alcohol, and betel quid and oral cancer: A prospective cohort study. J. Oncol..

[152] Nosratzehi T. (2017). Salivary Chemical Factors in Relation with Oral Cancer in Smokers and Non-Smokers: A Literature Review. J. Dent..

[153] Valavanidis A., Vlachogianni T., Fiotakis K. (2009). Tobacco smoke: Involvement of reactive oxygen species and stable free radicals in mechanisms of oxidative damage, carcinogenesis and synergistic effects with other respirable particles. Int. J. Environ. Res. Public Health.

[154] Sharifi-Rad M., Anil Kumar N.V., Zucca P., Varoni E.M., Dini L., Panzarini E., Rajkovic J., Tsouh Fokou P.V., Azzini E., Peluso I. (2020). Lifestyle, Oxidative Stress, and Antioxidants: Back and Forth in the Pathophysiology of Chronic Diseases. Front. Physiol..

[155] Chen C.L., Chi C.W., Liu T.Y. (2002). Hydroxyl radical formation and oxidative DNA damage induced by areca quid in vivo. J. Toxicol. Environ. Health Part A.

[156] Xu Z., Huang C.M., Shao Z., Zhao X.P., Wang M., Yan T.L., Zhou X.C., Jiang E.H., Liu K., Shang Z.J. (2017). Autophagy Induced by Areca Nut Extract Contributes to Decreasing Cisplatin Toxicity in Oral Squamous Cell Carcinoma Cells: Roles of Reactive Oxygen Species/AMPK Signaling. Int. J. Mol. Sci..

[157] Lu H.H., Kao S.Y., Liu T.Y., Liu S.T., Huang W.P., Chang K.W., Lin S.C. (2010). Areca nut extract induced oxidative stress and upregulated hypoxia inducing factor leading to autophagy in oral cancer cells. Autophagy.

[158] Radisky D.C., Levy D.D., Littlepage L.E., Liu H., Nelson C.M., Fata J.E., Leake D., Godden E.L., Albertson D.G., Nieto M.A. (2005). Rac1b and reactive oxygen species mediate MMP-3-induced EMT and genomic instability. Nature.

[159] Lee S.S., Tsai C.H., Yu C.C., Chang Y.C. (2013). Elevated snail expression mediates tumor progression in areca quid chewing-associated oral squamous cell carcinoma via reactive oxygen species. PLoS ONE.

[160] Lin M.H., Hsieh W.F., Chiang W.F., Hong W.Z., Hsu Y.R., Cheng Y.C., Chen T.C., Hsu K.C., Lin P.Y., Liu S.Y. (2010). Autophagy induction by the 30–100kDa fraction of areca nut in both normal and malignant cells through reactive oxygen species. Oral Oncol..

[161] Seitz H.K., Stickel F. (2007). Molecular mechanisms of alcohol-mediated carcinogenesis. Nat. Rev. Cancer.

[162] Cao J.Y., Mansouri S., Frappier L. (2012). Changes in the nasopharyngeal carcinoma nuclear proteome induced by the EBNA1 protein of Epstein-Barr virus reveal potential roles for EBNA1 in metastasis and oxidative stress responses. J. Virol..

[163] Lai D., Tan C.L., Gunaratne J., Quek L.S., Nei W., Thierry F., Bellanger S. (2013). Localization of HPV-18 E2 at mitochondrial membranes induces ROS release and modulates host cell metabolism. PLoS ONE.

[164] Williams V.M., Filippova M., Filippov V., Payne K.J., Duerksen-Hughes P. (2014). Human papillomavirus type 16 E6* induces oxidative stress and DNA damage. J. Virol..

[165] Marullo R., Werner E., Zhang H., Chen G.Z., Shin D.M., Doetsch P.W. (2015). HPV16 E6 and E7 proteins induce a chronic oxidative stress response via NOX2 that causes genomic instability and increased susceptibility to DNA damage in head and neck cancer cells. Carcinogenesis.

[166] Li L., Chen Y., Gibson S.B. (2013). Starvation-induced autophagy is regulated by mitochondrial reactive oxygen species leading to AMPK activation. Cell. Signal..

[167] Gao L., Dou Z.C., Ren W.H., Li S.M., Liang X., Zhi K.Q. (2019). CircCDR1as upregulates autophagy under hypoxia to promote tumor cell survival via AKT/ERK(1/2)/mTOR signaling pathways in oral squamous cell carcinomas. Cell Death Dis..

[168] Philibert R., Erwin C. (2015). A Review of Epigenetic Markers of Tobacco and Alcohol Consumption. Behav. Sci. Law.

[169] Chen J., Hutchison K.E., Bryan A.D., Filbey F.M., Calhoun V.D., Claus E.D., Lin D., Sui J., Du Y., Liu J. (2018). Opposite Epigenetic Associations With Alcohol Use and Exercise Intervention. Front. Psychiatry.

[170] Sabi S.H., Khabour O.F., Alzoubi K.H., Cobb C.O., Eissenberg T. (2020). Changes at global and site-specific DNA methylation of MLH1 gene promoter induced by waterpipe smoking in blood lymphocytes and oral epithelial cells. Inhal. Toxicol..

[171] Das D., Ghosh S., Maitra A., Biswas N.K., Panda C.K., Roy B., Sarin R., Majumder P.P. (2019). Epigenomic dysregulation-mediated alterations of key biological pathways and tumor immune evasion are hallmarks of gingivo-buccal oral cancer. Clin. Epigenetics.

[172] Friedman R.C., Farh K.K., Burge C.B., Bartel D.P. (2009). Most mammalian mRNAs are conserved targets of microRNAs. Genome Res..

[173] Behm-Ansmant I., Rehwinkel J., Izaurralde E. (2006). MicroRNAs silence gene expression by repressing protein expression and/or by promoting mRNA decay. Cold Spring Harb. Symp. Quant. Biol..

[174] Irimie A.I., Zimta A.A., Ciocan C., Mehterov N., Dudea D., Braicu C., Berindan-Neagoe I. (2018). The Unforeseen Non-Coding RNAs in Head and Neck Cancer. Genes.

[175] Tomuleasa C., Braicu C., Irimie A., Craciun L., Berindan-Neagoe I. (2014). Nanopharmacology in translational hematology and oncology. Int. J. Nanomed..

[176] Nagadia R., Pandit P., Coman W.B., Cooper-White J., Punyadeera C. (2013). miRNAs in head and neck cancer revisited. Cell. Oncol..

[177] Chu A., Robertson G., Brooks D., Mungall A.J., Birol I., Coope R., Ma Y., Jones S., Marra M.A. (2016). Large-scale profiling of microRNAs for The Cancer Genome Atlas. Nucleic Acids Res..

[178] Irimie A.I., Braicu C., Sonea L., Zimta A.A., Cojocneanu-Petric R., Tonchev K., Mehterov N., Diudea D., Buduru S., Berindan-Neagoe I. (2017). A Looking-Glass of Non-coding RNAs in oral cancer. Int. J. Mol. Sci..

[179] Alexander-Dann B., Pruteanu L.L., Oerton E., Sharma N., Berindan-Neagoe I., Módos D., Bender A. (2018). Developments in toxicogenomics: Understanding and predicting compound-induced toxicity from gene expression data. Mol. Omics.

[180] Troiano G., Boldrup L., Ardito F., Gu X., Lo Muzio L., Nylander K. (2016). Circulating miRNAs from blood, plasma or serum as promising clinical biomarkers in oral squamous cell carcinoma: A systematic review of current findings. Oral Oncol..

[181] Guo X., Lv X., Lv X., Ma Y., Chen L., Chen Y. (2017). Circulating miR-21 serves as a serum biomarker for hepatocellular carcinoma and correlated with distant metastasis. Oncotarget.

[182] Osan C., Chira S., Nutu A.M., Braicu C., Baciut M., Korban S.S., Berindan-Neagoe I. (2021). The Connection between MicroRNAs and Oral Cancer Pathogenesis: Emerging Biomarkers in Oral Cancer Management. Genes.

[183] Irimie A.I., Sonea L., Jurj A., Mehterov N., Zimta A.A., Budisan L., Braicu C., Berindan-Neagoe I. (2017). Future trends and emerging issues for nanodelivery systems in oral and oropharyngeal cancer. Int. J. Nanomed..

[184] Bersani C., Mints M., Tertipis N., Haeggblom L., Näsman A., Romanitan M., Dalianis T., Ramqvist T. (2018). MicroRNA-155, -185 and -193b as biomarkers in human papillomavirus positive and negative tonsillar and base of tongue squamous cell carcinoma. Oral Oncol..

[185] Fang C., Li Y. (2019). Prospective applications of microRNAs in oral cancer. Oncol. Lett..

[186] Wu M., Duan Q., Liu X., Zhang P., Fu Y., Zhang Z., Liu L., Cheng J., Jiang H. (2020). MiR-155-5p promotes oral cancer progression by targeting chromatin remodeling gene ARID2. Biomed. Pharmacother..

[187] Manikandan M., Deva Magendhra Rao A.K., Rajkumar K.S., Rajaraman R., Munirajan A.K. (2015). Altered levels of miR-21, miR-125b-2*, miR-138, miR-155, miR-184, and miR-205 in oral squamous cell carcinoma and association with clinicopathological characteristics. J. Oral Pathol. Med..

[188] Lajer C.B., Nielsen F.C., Friis-Hansen L., Norrild B., Borup R., Garnæs E., Rossing M., Specht L., Therkildsen M.H., Nauntofte B. (2011). Different miRNA signatures of oral and pharyngeal squamous cell carcinomas: A prospective translational study. Br. J. Cancer.

[189] Lopes C.B., Magalhães L.L., Teófilo C.R., Alves A., Montenegro R.C., Negrini M., Ribeiro-Dos-Santos Â. (2018). Differential expression of hsa-miR-221, hsa-miR-21, hsa-miR-135b, and hsa-miR-29c suggests a field effect in oral cancer. BMC Cancer.

[190] Jia B., Zhang S., Wu S., Zhu Q., Li W. (2021). MiR-770 promotes oral squamous cell carcinoma migration and invasion by regulating the Sirt7/Smad4 pathway. IUBMB Life.

[191] Rajan C., Roshan V.G.D., Khan I., Manasa V.G., Himal I., Kattoor J., Thomas S., Kondaiah P., Kannan S. (2021). MiRNA expression profiling and emergence of new prognostic signature for oral squamous cell carcinoma. Sci. Rep..

[192] Kim J.S., Yu S.K., Lee M.H., Park M.G., Park E., Kim S.G., Lee S.Y., Kim C.S., Kim H.J., Chun H.S. (2013). MicroRNA-205 directly regulates the tumor suppressor, interleukin-24, in human KB oral cancer cells. Mol. Cells.

[193] Kozaki K., Imoto I., Mogi S., Omura K., Inazawa J. (2008). Exploration of tumor-suppressive microRNAs silenced by DNA hypermethylation in oral cancer. Cancer Res..

[194] Uesugi A., Kozaki K., Tsuruta T., Furuta M., Morita K., Imoto I., Omura K., Inazawa J. (2011). The tumor suppressive microRNA miR-218 targets the mTOR component Rictor and inhibits AKT phosphorylation in oral cancer. Cancer Res..

[195] Shiah S.G., Hsiao J.R., Chang W.M., Chen Y.W., Jin Y.T., Wong T.Y., Huang J.S., Tsai S.T., Hsu Y.M., Chou S.T. (2014). Downregulated miR329 and miR410 promote the proliferation and invasion of oral squamous cell carcinoma by targeting Wnt-7b. Cancer Res..

[196] Childs G., Fazzari M., Kung G., Kawachi N., Brandwein-Gensler M., McLemore M., Chen Q., Burk R.D., Smith R.V., Prystowsky M.B. (2009). Low-level expression of microRNAs let-7d and miR-205 are prognostic markers of head and neck squamous cell carcinoma. Am. J. Pathol..

[197] Kao Y.Y., Chou C.H., Yeh L.Y., Chen Y.F., Chang K.W., Liu C.J., Fan Chiang C.Y., Lin S.C. (2019). MicroRNA miR-31 targets SIRT3 to disrupt mitochondrial activity and increase oxidative stress in oral carcinoma. Cancer Lett..

[198] Endo H., Muramatsu T., Furuta M., Uzawa N., Pimkhaokham A., Amagasa T., Inazawa J., Kozaki K. (2013). Potential of tumor-suppressive miR-596 targeting LGALS3BP as a therapeutic agent in oral cancer. Carcinogenesis.

[199] Bhat M.Y., Advani J., Rajagopalan P., Patel K., Nanjappa V., Solanki H.S., Patil A.H., Bhat F.A., Mathur P.P., Nair B. (2018). Cigarette smoke and chewing tobacco alter expression of different sets of miRNAs in oral keratinocytes. Sci. Rep..

[200] Shiah S.G., Hsiao J.R., Chang H.J., Hsu Y.M., Wu G.H., Peng H.Y., Chou S.T., Kuo C.C., Chang J.Y. (2020). MiR-30a and miR-379 modulate retinoic acid pathway by targeting DNA methyltransferase 3B in oral cancer. J. Biomed. Sci..

[201] Peng H.Y., Hsiao J.R., Chou S.T., Hsu Y.M., Wu G.H., Shieh Y.S., Shiah S.G. (2020). MiR-944/CISH mediated inflammation via STAT3 is involved in oral cancer malignance by cigarette smoking. Neoplasia.

[202] Arunkumar G., Deva Magendhra Rao A.K., Manikandan M., Prasanna Srinivasa Rao H., Subbiah S., Ilangovan R., Murugan A.K., Munirajan A.K. (2018). Dysregulation of miR-200 family microRNAs and epithelial-mesenchymal transition markers in oral squamous cell carcinoma. Oncol. Lett..

[203] Krishnan A.R., Zheng H., Kwok J.G., Qu Y., Zou A.E., Korrapati A., Li P.X., Califano J.A., Hovell M.F., Wang-Rodriguez J. (2017). A comprehensive study of smoking-specific microRNA alterations in head and neck squamous cell carcinoma. Oral Oncol..

[204] Avissar M., McClean M.D., Kelsey K.T., Marsit C.J. (2009). MicroRNA expression in head and neck cancer associates with alcohol consumption and survival. Carcinogenesis.

[205] Manikandan M., Deva Magendhra Rao A.K., Arunkumar G., Rajkumar K.S., Rajaraman R., Munirajan A.K. (2015). Down Regulation of miR-34a and miR-143 May Indirectly Inhibit p53 in Oral Squamous Cell Carcinoma: A Pilot Study. Asian Pac. J. Cancer Prev..

[206] Chou S.T., Peng H.Y., Mo K.C., Hsu Y.M., Wu G.H., Hsiao J.R., Lin S.F., Wang H.D., Shiah S.G. (2019). MicroRNA-486-3p functions as a tumor suppressor in oral cancer by targeting DDR1. J. Exp. Clin. Cancer Res..

[207] Tsai Y.S., Lin C.S., Chiang S.L., Lee C.H., Lee K.W., Ko Y.C. (2011). Areca nut induces miR-23a and inhibits repair of DNA double-strand breaks by targeting FANCG. Toxicol. Sci..

[208] Chuerduangphui J., Ekalaksananan T., Chaiyarit P., Patarapadungkit N., Chotiyano A., Kongyingyoes B., Promthet S., Pientong C. (2018). Effects of arecoline on proliferation of oral squamous cell carcinoma cells by dysregulating c-Myc and miR-22, directly targeting oncostatin M. PLoS ONE.

[209] Singh P., Srivastava A.N., Sharma R., Mateen S., Shukla B., Singh A., Chandel S. (2018). Circulating MicroRNA-21 Expression as a Novel Serum Biomarker for Oral Sub-Mucous Fibrosis and Oral Squamous Cell Carcinoma. Asian Pac. J. Cancer Prev..

[210] Cao M.X., Zhang W.L., Yu X.H., Wu J.S., Qiao X.W., Huang M.C., Wang K., Wu J.B., Tang Y.J., Jiang J. (2020). Interplay between cancer cells and M2 macrophages is necessary for miR-550a-3-5p down-regulation-mediated HPV-positive OSCC progression. J. Exp. Clin. Cancer Res..

[211] Božinović K., Sabol I., Dediol E., Milutin Gašperov N., Manojlović S., Vojtechova Z., Tachezy R., Grce M. (2019). Genome-wide miRNA profiling reinforces the importance of miR-9 in human papillomavirus associated oral and oropharyngeal head and neck cancer. Sci. Rep..

[212] Zhang Q., Chen Y., Hu S.Q., Pu Y.M., Zhang K., Wang Y.X. (2020). A HPV16-related prognostic indicator for head and neck squamous cell carcinoma. Ann. Transl. Med..

[213] House R., Majumder M., Janakiraman H., Ogretmen B., Kato M., Erkul E., Hill E., Atkinson C., Barth J., Day T.A. (2018). Smoking-induced control of miR-133a-3p alters the expression of EGFR and HuR in HPV-infected oropharyngeal cancer. PLoS ONE.

[214] Lepore S., Lettini G., Condelli V., Sisinni L., Piscazzi A., Simeon V., Zoppoli P., Pedicillo M.C., Natalicchio M.I., Pietrafesa M. (2020). Comparative Gene Expression Profiling of Tobacco-Associated HPV-Positive versus Negative Oral Squamous Carcinoma Cell Lines. Int. J. Med. Sci..

[215] Sacconi A., Donzelli S., Pulito C., Ferrero S., Spinella F., Morrone A., Rigoni M., Pimpinelli F., Ensoli F., Sanguineti G. (2020). TMPRSS2, a SARS-CoV-2 internalization protease is downregulated in head and neck cancer patients. J. Exp. Clin. Cancer Res..

[216] Cai L., Ye Y., Jiang Q., Chen Y., Lyu X., Li J., Wang S., Liu T., Cai H., Yao K. (2015). Epstein-Barr virus-encoded microRNA BART1 induces tumour metastasis by regulating PTEN-dependent pathways in nasopharyngeal carcinoma. Nat. Commun..

[217] Cai L., Long Y., Chong T., Cai W., Tsang C.M., Zhou X., Lin Y., Ding T., Zhou W., Zhao H. (2019). EBV-miR-BART7-3p Imposes Stemness in Nasopharyngeal Carcinoma Cells by Suppressing SMAD7. Front. Genet..

[218] Taş B., Güre A.O. (2020). The effect of Maras powder and smoking on the microRNA deregulation of oral mucosa. J. Appl. Oral Sci. Rev. FOB.

[219] Rishabh K., Khadilkar S., Kumar A., Kalra I., Kumar A.P., Kunnumakkara A.B. (2021). MicroRNAs as Modulators of Oral Tumorigenesis-A Focused Review. Int. J. Mol. Sci..

[220] Paluszczak J. (2020). The Significance of the Dysregulation of Canonical Wnt Signaling in Head and Neck Squamous Cell Carcinomas. Cells.

[221] Ye Y., Zhou Y., Zhang L., Chen Y., Lyu X., Cai L., Lu Y., Deng Y., Wang J., Yao K. (2013). EBV-miR-BART1 is involved in regulating metabolism-associated genes in nasopharyngeal carcinoma. Biochem. Biophys. Res. Commun..

